# Beyond Sequence Optimization in Enzyme Engineering: Protein Maturation and Operational Stability

**DOI:** 10.3390/ijms27219682

**Published:** 2026-10-29

**Authors:** Suejeong Paik, Sang-Woo Han

**Affiliations:** Department of Biotechnology, College of Biomedical & Health Science, Konkuk University, Chungju 27478, Chungbuk, Republic of Korea

**Keywords:** enzyme engineering, protein maturation, catalytic competence, post-translational modification, proteoform, conformational dynamics, operational stability

## Abstract

Enzyme engineering commonly focuses on catalytic performance and recombinant protein production. Neither focus alone reveals how much expressed protein becomes an active catalyst or how long it remains functional. This review discusses engineering strategies for catalyst formation and maintenance using four representative enzyme groups. Cytochrome P450 enzymes illustrate the roles of cofactor incorporation and redox-system organization. Zymogenic proteases show how precursor maturation and autolysis control the active-enzyme population. Fungal cellulases demonstrate how post-translational modifications and host processing shape the final proteoform. Lytic polysaccharide monooxygenases (LPMOs) link active-site maturation to protection against oxidative damage. Together, these examples point to common strategies for improving catalyst formation and catalytic lifetime. Emerging approaches extend these principles to accessory maturation machinery and controlled activation. Conformational engineering and state-resolved measurements provide further opportunities for enzyme design.

## 1. Introduction

Sequence optimization by introducing mutations remains central to enzyme engineering because catalytic residues and substrate-binding regions are encoded by the amino-acid sequence, which also strongly influences protein stability. However, the sequence of a catalytic enzyme does not by itself describe the enzyme population obtained after recombinant expression. An expressed polypeptide must pass through folding and maturation before it can contribute to catalysis, and then the resulting active enzyme must remain functional during catalysis. These additional steps become critical when recombinant protein abundance and catalytic performance are not proportional. In such cases, sequence improvement can be limited by processes that occur before the active catalyst forms or by processes that remove it from productive turnover after formation.

The first limitation is the conversion of expressed protein into a catalytically competent form. Cofactor-dependent enzymes make this distinction especially clear because apoenzyme and holoenzyme can coexist within a single recombinant preparation. Studies of CYP3A4 and CYP2D6 have shown that changing cellular heme allocation can alter the amount of incorporated heme and the resulting catalytic activity, with comparatively small changes in total P450 abundance [1]. A complementary whole-cell study reached the same conclusion by quantifying holo-P450 formation after engineering heme supply [2]. Precursor-dependent proteases illustrate the same principle through a different mechanism. Folding and proteolytic maturation determine their final activity, so accumulation of recombinant precursor does not necessarily indicate accumulation of active enzyme [3,4]. Together, these examples place protein maturation between sequence-level expression and catalytic performance.

Post-translational modification (PTM) adds another layer because a single encoded sequence can give rise to multiple proteoforms. Fungal cellulases illustrate this well. Expression of the same cellobiohydrolase in different yeast hosts yielded distinct glycosylated forms with different physical and thermal properties [5], and site-specific studies have shown that individual glycans can alter local structure and catalytic behavior [6,7]. A comparable host-dependent effect has been reported for glycosylated D-lactonohydrolase produced in *Pichia pastoris* [8]. Therefore, the production host is not merely a source of recombinant protein but part of the biochemical environment that determines the final catalyst.

A second limitation appears after maturation. An enzyme can be correctly formed but lose activity during catalysis. Proteases can undergo autolysis [9], lignocellulolytic enzymes can be sequestered by nonproductive surfaces [10], and reactive metalloenzymes can damage their own active sites during turnover [11]. Such processes shift the engineering question from how much active enzyme is generated to how long that enzyme remains available for productive catalysis.

Lytic polysaccharide monooxygenases (LPMOs) bring the two limitations together within a single catalytic system. Precise N-terminal processing is required to form the histidine-brace copper site, and the exposed N-terminal H1 can then undergo a host-dependent methylation that is coupled to that processing [12,13]. Once the copper center is assembled, the oxidative chemistry that cleaves polysaccharides can also drive self-damage when substrate engagement is poor [11]. LPMOs provide an integrated example, in which active-site formation and preservation of that site during turnover can be examined within the same molecular framework.

**Figure 1:**
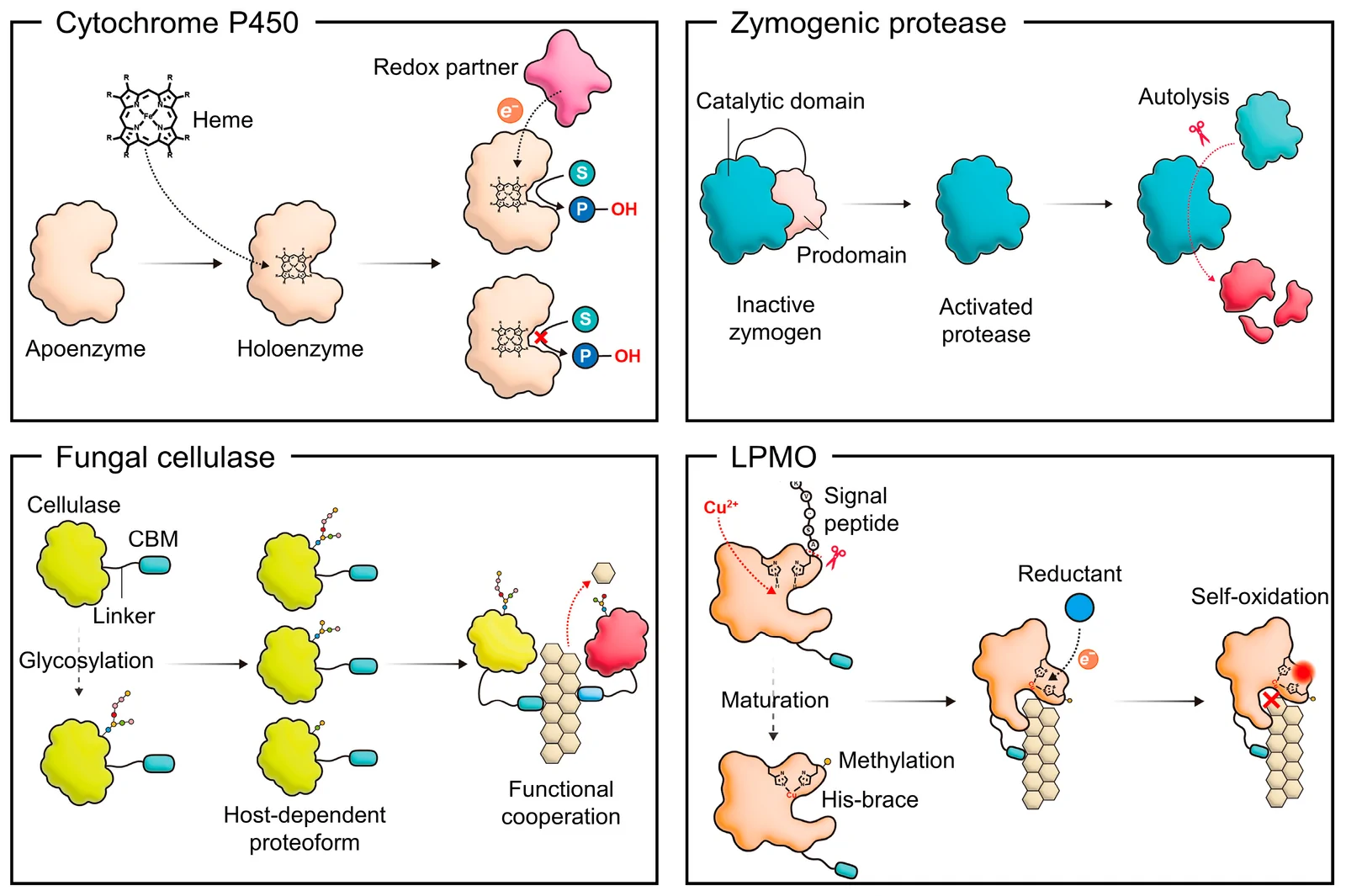
Schematic comparison of the molecular processes that form and maintain functional catalysts in four representative enzyme classes. In cytochrome P450s, heme incorporation forms the holoenzyme, while redox-partner interactions determine productive coupling or uncoupling. In zymogenic proteases, prodomain cleavage activates the zymogen, whereas autolysis causes self-digestion of the mature enzyme. In fungal cellulases, host-dependent site-specific post-translational modifications (PTMs) generate distinct proteoforms, whose modular organization supports coordinated enzyme function. In lytic polysaccharide monooxygenases (LPMOs), signal-peptide removal exposes the N-terminal histidine (H1), and copper loading establishes the histidine-brace active site. Histidine methylation can protect fungal LPMOs from oxidative damage. Productive turnover requires reduction in the copper center, whereas off-pathway oxidative chemistry can cause oxidative inactivation by damaging the histidine brace.

This review uses these enzyme classes to examine how protein maturation and operational stability can be incorporated into enzyme engineering without replacing sequence optimization as the central design tool. We first define the experimental distinction between producing recombinant protein and producing functional enzyme, then treat cofactor assembly, precursor maturation, and host-dependent proteoform generation as separate problems. We use LPMOs to show how these processes can converge at a reactive active site and then discuss emerging strategies for controlling enzyme maturation and function through protein engineering and data-guided design. Throughout this review, we focus on studies that directly measure how an engineering intervention alters the biochemical or structural properties of an enzyme and how those changes affect catalytic function (Figure 1). Rather than treating enzyme maturation and stability as separate engineering objectives, this review examines how engineering affects catalyst formation, productive turnover, and catalytic persistence across four enzyme classes. We distinguish direct evidence of changes in functional enzyme states from indirect measures of performance, providing a common framework for interpreting engineering outcomes and identifying the limitations of existing studies. The literature search covered PubMed, Europe PMC, and Scopus, focusing primarily on original research published between 2021 and 2026 while including earlier studies that established key mechanistic concepts. Study selection prioritized relevant engineering strategies and mechanistic evidence for catalyst formation and maintenance.

## 2. From Recombinant Protein to Functional Biocatalyst

Recombinant expression produces protein, but catalytic performance reflects only the fraction that reaches and remains in a functional form. Before catalysis, an expressed polypeptide may require cofactor incorporation or precursor processing. Host-dependent modification can further shape its final functional state. The resulting enzyme can then lose useful activity through self-inactivation, structural damage, or nonproductive interactions. Enzyme engineering addresses two related but experimentally separable problems. The first is formation of a catalytically competent enzyme. The second is maintenance of that enzyme during catalysis. The formed enzyme must also reach a productive molecular state. Redox-partner organization or substrate binding can change turnover without changing the amount of active enzyme. These distinctions separate catalyst formation from productive turnover and initial performance from catalytic lifetime.

### 2.1. Acquisition of Catalytic Competence

The first stage concerns the conversion of expressed protein into a form capable of catalysis. The molecular requirement differs among enzyme classes. In P450s, heme incorporation determines how much of the expressed protein becomes holoenzyme. Zymogenic proteases must complete precursor-dependent folding and proteolytic maturation, whereas fungal cellulases can acquire host-dependent PTMs that generate functionally distinct proteoforms. LPMOs combine several of these requirements because establishing the catalytic center depends on both correct post-translational maturation and copper loading [2,4,7,13].

Although the underlying processes differ, they lead to the same experimental problem. Total recombinant protein does not necessarily represent the amount of catalytically competent enzymes. Different measurements can address this problem. Holoenzyme fraction can report cofactor incorporation, precursor analysis can show proteolytic maturation, and mass spectrometry can resolve site-specific PTMs and distinct proteoforms. These complementary measurements also help determine which stage of enzyme function is affected by an engineering strategy. Increased specific activity or product formation indicates improved performance, but this improvement may arise from a higher proportion of active enzymes, faster catalytic turnover, or both. Therefore, establishing improved maturation requires direct measurement of the molecular changes targeted by the engineering strategy. In the following sections, we distinguish studies that directly demonstrate changes in enzyme maturation from those that infer such changes from activity measurements alone.

### 2.2. Maintenance of Catalytic Activity Under Operating Conditions

Catalytic competence does not ensure that an enzyme will remain active throughout a reaction. Even after the active form has been generated, the active fraction can decline as the reaction proceeds. Proteases can be degraded through autolysis, lignocellulolytic enzymes can lose activity through nonproductive adsorption, and reactive metalloenzymes can undergo chemical damage driven by their own catalytic chemistry [9,10,11]. These losses determine operational stability, which describes the ability of an enzyme to maintain catalytic activity under reaction conditions. Stability measured during storage or after heat treatment may not predict this behavior because substrate interactions and catalytic turnover can cause additional inactivation.

Evaluating this stage requires measurements that distinguish loss of active enzyme from changes in intrinsic catalytic properties, such as residual activity, active-site integrity, or the fraction of active enzyme. Combined with catalytic readouts, these measurements make it possible to determine whether an engineering strategy increases the amount of active enzyme initially produced, improves the catalytic properties of the enzyme, or extends the period over which it remains functional. Therefore, operational half-life and time-dependent activity profiles provide more direct information on catalytic persistence than a single endpoint activity measurement. Table 1 summarizes the measurements used to distinguish these functional stages.

**Figure 2:**
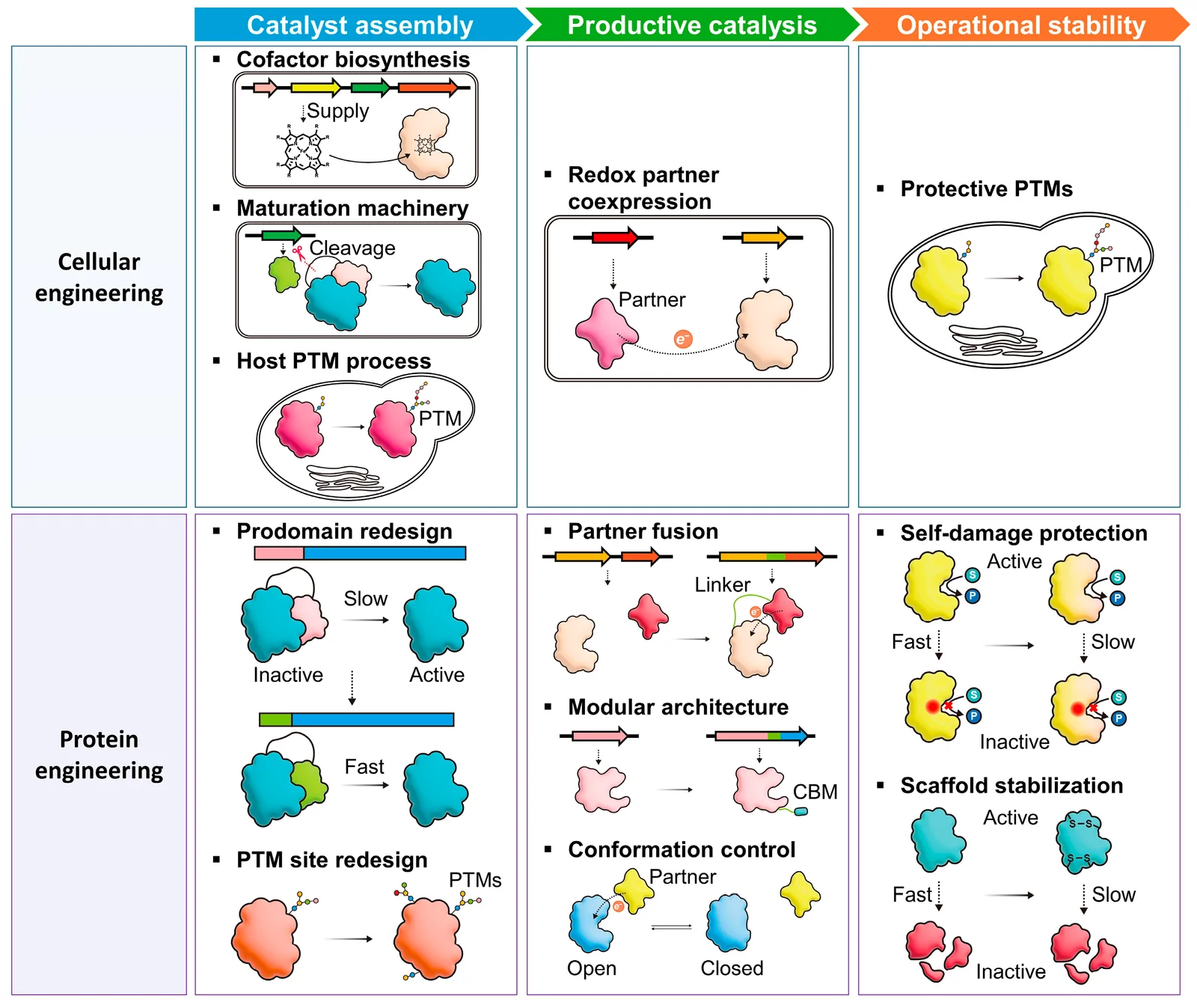
Engineering strategies for controlling functional enzyme states at the cellular and protein levels. Strategies are organized according to their primary engineering targets, including catalyst assembly, productive catalysis, and operational stability. Cellular engineering can increase catalyst formation by improving cofactor supply or accessory maturation machinery. Host PTM pathways can further shape the properties of the resulting proteoform. Productive catalysis can be improved by co-expressing compatible redox partners, while protective PTMs can increase resistance to inactivation. Protein engineering can improve catalyst assembly through prodomain or cleavage-site redesign and by modifying PTM sites. Productive catalysis can be improved by facilitating electron transfer through redox-partner fusion, tuning substrate-facing architecture, or favoring productive conformational states. Operational stability can be increased by introducing mutations that reduce autolysis or oxidative self-damage and by installing stabilizing covalent constraints such as engineered disulfide bonds.

The following sections examine how each of these stages can limit enzyme performance. P450s illustrate how cofactor loading and redox coupling affect catalytic function. Zymogenic proteases show how precursor maturation and self-inactivation limit the active-enzyme population. Fungal cellulases demonstrate how host-dependent PTMs and noncatalytic architecture shape enzyme properties. LPMOs integrate these processes at a single copper center, where active-site formation, substrate engagement, and oxidative damage are closely linked. These functional stages can be addressed through cellular- and protein-level engineering strategies (Figure 2).

## 3. Cofactor-Defined Catalyst Formation in Cytochrome P450s

Cytochrome P450 monooxygenases provide a clear example of why recombinant protein production does not directly indicate catalytic competence. An active P450 requires both proper heme incorporation and productive electron transfer through a compatible redox system. These requirements define two engineering stages. Heme incorporation controls holoenzyme formation, whereas redox-system organization controls electron delivery to the heme and coupling to substrate oxidation. In CYP3A4 and CYP2D6, changes in cellular heme allocation altered heme incorporation and catalytic activity [1]. Nitric oxide shifted the balance between heme incorporation and heme loss in a concentration-dependent manner [19]. Together, these studies separate catalyst formation from productive turnover.

### 3.1. Engineering Holoenzyme Formation

Heme supply can limit holo-P450 formation even when P450 expression is high. Hu et al. tested whether increasing endogenous heme biosynthesis could raise the fraction of expressed P450 that binds heme in *Escherichia coli* C41(DE3) [2]. They integrated additional copies of the *E. coli hemA*, *hemL*, *hemB*, *hemC*, *hemD*, and *hemH* genes at selected ratios and assembled the rate-limiting enzymes HemB, HemC, and HemD on DNA-guided scaffolds to increase pathway flux. This strategy increased the holo fraction from 17.7% to 71.5% for BM3mut and from 12.9% to 61.8% for sca-2mut. CYP105D7 showed a smaller increase from 6.8% to 12.2%. The increase in holo-P450 despite similar P450 expression shows that intracellular heme supply can directly limit the amount of functional enzyme. Heme supply can also complement improvements made to the P450 sequence itself. Yu et al. first used directed evolution to improve a self-sufficient CYP153A-NCP enzyme for terminal hydroxylation of lauric acid [20]. The selected R14R/D629G variant showed higher activity, and analysis of D629G linked the improvement to faster turnover and a shorter predicted flavin adenine dinucleotide (FAD)–flavin mononucleotide (FMN) electron-transfer distance. The authors then strengthened heme biosynthesis by increasing *hemB* expression and deleting the *yfeX* gene involved in heme degradation. The resulting heme-pathway modification further increased ω-aminododecanoic acid production by 17.3% relative to the strain carrying the same P450 variant without heme-pathway engineering. These studies show that P450 formation involves more than protein expression. Heme availability determines how much expressed protein can reach the holoenzyme state. A simpler approach is to supplement the culture medium with 5-aminolevulinic acid (5-ALA) to improve heme availability during recombinant P450 production [21]. Chang et al. evaluated the effect of 5-ALA supplementation on a fungal P450 expressed in *E. coli* [22]. Adding 0.5 mM 5-ALA increased 14α-hydroxyprogesterone production to 12.7 mg/L, whereas production remained very low without supplementation. This increase in product formation supports improved whole-cell performance, although the extent of holo-P450 formation was not directly measured. Kato et al. developed an alternative strategy by introducing a heterologous C4 pathway into *E. coli* [23]. They expressed 5-aminolevulinic acid synthase from *Paracoccus denitrificans* to increase intracellular 5-ALA production from glycine and succinyl-CoA. The engineered strain accumulated more intracellular heme than cells supplemented with exogenous 5-ALA and improved holoenzyme production. Together with the heme-pathway engineering studies described above, these results show that heme availability can be improved through precursor supplementation or metabolic engineering.

### 3.2. Engineering Productive Electron Transfer and Coupling

A properly formed holoenzyme still requires a compatible redox chain. Partner identity and native protein architecture can limit this step. Sun et al. compared full-length human CYP2E1 with an N-terminally truncated form commonly used for recombinant expression [24]. The full-length enzyme associated more strongly with NADPH-cytochrome P450 oxidoreductase (POR) and cytochrome b_5_. Its apparent *K*_M_ for POR decreased from 1.2 to 0.36 μM, and catalytic turnover increased. Koroleva et al. used transcriptomic analysis and biochemical reconstitution to identify Fdx4 and FdR3 as native redox partners of DoxA in *Streptomyces peucetius* [25]. Additional expression of *fdx4* and *fdr3* increased doxorubicin production by 1.8-fold. Additional *doxA* expression alone had little effect. These examples show that increasing P450 abundance cannot compensate for weak or mismatched electron-delivery interactions.

Redox-partner compatibility also reflects the chemical properties of the partner. Urban et al. altered the redox properties of cytochrome b_5_ while preserving its protein scaffold by replacing the native heme with porphyrin analogs [26]. Iron-mesoporphyrin lowered the redox potential by approximately 30 mV. Zinc-protoporphyrin generated a redox-inactive form. CYP3A4 stimulation followed the redox capability of cytochrome b_5_. These findings show that productive P450 catalysis requires a compatible electron donor with suitable redox properties.

Once compatible redox modules are identified, covalent fusion can improve their spatial organization. A foundational study fused the biosynthetic P450 PikC to the RhFRED reductase domain [27]. This produced a single-component self-sufficient catalyst and established heterologous reductase fusion as a P450 engineering strategy. Xiang et al. used the same design for the avermectin-oxidizing P450-Ema1 M212A variant by fusing it to the reductase domain of P450RhF [28]. Linker optimization yielded the F4 fusion with a 9-residue linker (GEPAARAVS). The fusion showed higher catalytic efficiency than the non-fused system and converted approximately 90% of avermectin to 4″-oxo-avermectin within 8 h. These studies show that covalent fusion can make redox-partner proximity a controllable design variable.

Protein architecture can define the route used for electron transfer between redox centers. The full-length structure of the self-sufficient CYP116B46 placed FMN, the [2Fe–2S] center, and heme along the direction of electron flow [29]. The [2Fe–2S]-to-heme edge-to-edge distance was approximately 25.3 Å. Mutational analysis identified residues that contribute to intramolecular electron transfer. Xu et al. used a sequence-level strategy in a tryptamine 5-hydroxylase (T5H)–cytochrome P450 reductase (CPR) system and predicted an electron-hopping pathway [30]. Screening identified H683F and V690F as aromatic relay mutations. The H683F/V690F reductase shortened the predicted route from nine hopping steps over 50.8 Å to six steps over 26 Å. Under identical reaction conditions, the mutant consumed 4.09-fold more NADPH over 120 min and increased T5H catalytic efficiency by approximately 22-fold. These studies show that short interdomain distance alone does not define productive electron transfer. The protein must provide a suitable electron-transfer route. In the T5H–CPR system, higher NADPH consumption supports improved reductant use, but it does not by itself quantify coupling. Product formation and NADPH consumption would need to be measured together to determine how much reducing power enters the desired reaction.

Domain motion can further influence electron transfer by changing the relative positions of the redox centers. Liang et al. studied the self-sufficient P450 chimera L21, which combines the heme domain of Vdh-K1 with the reductase and ferredoxin domains of CYP116B46 [17]. Semi-rational engineering at the heme–ferredoxin interface identified F346K/R354M, which facilitated a transition of the ferredoxin domain from a distal to a proximal conformation. Computational analysis showed that the transition barrier decreased from approximately 6 to 1.5 kcal/mol. The predicted [2Fe–2S]-to-heme electron-transfer pathway shortened from 32.36 to 28.31 Å, and the calculated electron-transfer rate increased by approximately 72-fold. Combining these mutations with active-site substitutions produced L21-M5 (P83A/A177M/K180F/F346K/R354M), which showed an 8.2-fold increase in catalytic efficiency and a coupling efficiency of 56.78%. The results link redox-domain motion to productive electron delivery.

Engineering multidomain assembly can also improve structural stability. Essert et al. used the SpyTag003-SpyCatcher003 system to covalently connect components of a chimeric P450 BM3 assembly [31]. The linked system was approximately twofold more active than the non-covalently associated assembly. It retained 80–90% of its initial activity after two months at 5 °C, and had a half-life exceeding two months at 20 °C. Giuriato et al. instead addressed linker-mediated stability in a P450SPα–sarcosine oxidase chimera [32]. They replaced an 11-residue glycine-rich flexible linker with an 11-residue proline-rich rigid linker. Differential Scanning Calorimetry (DSC) analysis of the flexible- and rigid-linker constructs showed unfolding enthalpies of approximately 561 and 765 kcal/mol, respectively. These thermodynamic measurements should not be interpreted as kinetic activation energies. The rigid-linker construct also showed a 5.7 °C increase in the heat-treatment-based *T*_50_ determined by CO-binding measurements, and lauric-acid conversion at 40 °C increased from 24% to 98%. These studies evaluated stability under different conditions. Essert et al. measured activity retention during prolonged storage [31], while Giuriato et al. examined thermal unfolding, heat-treatment resistance, and substrate conversion at 40 °C [32]. The reaction systems also differ, as the P450SPα–SOX fusion uses SOX-generated H_2_O_2_ rather than the NADPH-dependent electron transfer of P450 BM3. Nevertheless, both studies show that modifying domain assembly can improve enzyme stability and catalytic performance without directly altering the P450 catalytic domain.

These studies identify distinct engineering targets after holoenzyme formation. Redox-partner compatibility influences electron delivery to the P450. Protein architecture influences the arrangement of redox centers, while domain motion can change their relative positions during catalysis. Stabilizing the assembled system can improve activity retention during storage or resistance to thermal treatment, although these measurements do not establish operational stability during prolonged catalysis. Increased product formation alone cannot identify which mechanism changed. Holoenzyme formation and redox-system engineering therefore represent different stages of P450 engineering. Heme availability influences the amount of functional holoenzyme that can form, whereas redox-system engineering affects productive electron transfer and may improve the stability of the assembled system. Separating these stages helps distinguish catalyst formation, productive catalysis, and activity retention.

## 4. Engineering Precursor Maturation and Mature-Enzyme Stability in Zymogenic Proteases

Zymogenic proteases shift the problem from cofactor assembly to precursor maturation. The prodomain and cleavage site control precursor folding and activation. Host processing machinery can impose a separate limit. Beta-lytic protease requires extracellular trans-processing [4]. *Aspergillus niger* PepA undergoes acid-triggered intramolecular cleavage [33]. Recombinant butelase-1 is activated through an acidic bimolecular pathway [34]. These mechanisms differ, but each requires efficient formation of the mature enzyme and preservation of the activated protease after formation.

The prodomain can act as an intramolecular chaperone rather than only as an inhibitory segment. In subtilisin, the propeptide templates folding of the protease domain [35]. A single propeptide mutation generated an altered but enzymatically active conformation of the same mature subtilisin sequence. Sequence outside the final mature domain can therefore determine the functional state of that domain. Prodomains can also prevent premature proteolysis until cleavage occurs. Inefficient folding or incomplete processing can thus limit mature active-enzyme production even when precursor expression is high.

Activation also creates a second problem because the mature protease can become susceptible to self-cleavage or other forms of inactivation. Engineering zymogenic proteases therefore involves two related issues. The first is efficient formation of the mature enzyme through precursor folding and processing. The second is maintenance of the mature catalytic state after activation. These stages can be addressed by engineering of the prodomain, cleavage sites, secretion and processing machinery, and residues that control the stability of the mature enzyme.

### 4.1. Engineering Precursor Folding and Activation

Precursor sequence can be engineered in both the prodomain and the cleavage site. Zhang et al. targeted both features in the *A. niger* aspartic protease PepA [33]. Replacing the P1 alanine at the autocleavage site with tryptophan increased enzyme activity by 39.97%. The authors separately engineered residues within a prodomain α-helix that interacts with the mature domain. F37Y increased activity by 92.53%. The combined L26G/L34Y/F37Y variant showed a 2.83-fold increase over the wild type. Structural modeling suggested that these mutations altered prodomain conformation and its interaction with the mature domain. A similar strategy was applied to a *Bacillus licheniformis* keratinase expressed in *Bacillus subtilis* WB600 [36]. Stepwise prodomain truncation identified structural elements required for efficient mature-enzyme formation. Substitutions at the P1 residue L105 showed that cleavage-site sequence affects mature-enzyme activity. Combining sequence comparison with site-directed mutagenesis, the authors identified beneficial prodomain residues. The selected variant 2-D12 contained mutations at six positions and reached a specific activity of 236.8 KU/mg, corresponding to 186% of the original enzyme. Together, these studies show that engineering the prodomain and cleavage region can improve protease activity through different modifications of the precursor sequence. Although both approaches increased enzyme activity, the reported improvements are not directly comparable because the studies used different enzymes and assay conditions. Moreover, activity measurements alone cannot determine whether these improvements result from more efficient precursor maturation or changes in the properties of the mature enzyme.

Host processing capacity can impose a further limit on mature-enzyme formation. Li et al. found that deletion of *sipA* caused the greatest accumulation of alkaline-protease AprE precursor among the tested signal-peptidase genes in *Bacillus amyloliquefaciens* [37]. An additional chromosomal copy of *sipA* increased extracellular AprE activity by 19.9%. Heterologous secretion can introduce other processing requirements. Tc-LysN from *Trametes coccinea* was expressed in *Komagataella phaffii* using the *Saccharomyces cerevisiae* α-mating-factor signal peptide, an architecture that requires Kex2-dependent processing of both the secretion leader and the native prodomain-catalytic domain junction [15]. Both the 38 kDa zymogen and 19.8 kDa mature enzyme accumulated in the medium, indicating incomplete precursor maturation under this secretion system. Together, these studies show that host-dependent processing can limit the production of mature proteases at different stages of secretion. Increasing *sipA* expression improved extracellular AprE activity, whereas the coexistence of precursor and mature Tc-LysN directly indicated incomplete maturation. Measuring the precursor-to-mature ratio together with active-site concentration would help determine whether increased extracellular activity results from more efficient maturation or improved secretion.

Precursor engineering and host processing target different aspects of protease maturation. Prodomain and cleavage-site mutations modify the precursor itself, whereas host engineering alters the machinery responsible for its processing. These strategies should therefore be evaluated using enzyme activity measurements together with direct evidence of precursor maturation, not extracellular activity increases alone.

### 4.2. Preserving and Measuring the Mature Active State

Proteolytic activation creates a second engineering problem because the mature enzyme can cleave itself once the protective prodomain has been removed. Niu et al. addressed precursor production and mature-enzyme self-degradation separately in a *Streptomyces fradiae* trypsin expressed in *K. phaffii* [9]. They first replaced the native prodomain with an artificial sequence consisting of thioredoxin A (TrxA), the bovine trypsinogen activation peptide (DDDDK), and the hydrophobic peptide (FVEF). This precursor redesign increased trypsin activity from 13.8 to 33.9 U/mL. The authors then identified potential autolytic cleavage sites within the mature enzyme and introduced the R295A/R315A substitutions to remove two susceptible arginine residues. The double mutant showed reduced self-degradation and increased activity to 58.86 U/mL. It also shifted the optimum pH from 9.0 to 9.5 and showed a significantly longer half-life at pH 10. Precursor engineering and mature-enzyme stabilization therefore address different limitations within the same protease system.

Mature-protease engineering also requires measurements that distinguish total protein from catalytically active enzyme. Morales Granda et al. illustrated this point in engineered mouse chymotrypsin B1 variants [14]. After activating purified chymotrypsinogen with trypsin, the authors estimated total protein from UV absorbance and determined active chymotrypsin concentration by titration with the high-affinity inhibitor ecotin. Residual activity across ecotin concentrations gave an x-intercept corresponding to the concentration of protease containing a functional active site. The authors used this titrated concentration for kinetic analysis. All recombinant constructs contained Y164A to suppress autolysis. The study did not compare Y164A with the autolysis-prone sequence, so it does not quantify stabilization by this mutation. Its main contribution here is the measurement strategy. Mature-size protein and total protein concentration do not necessarily equal catalytically functional protease concentration.

These studies show that mature-protease engineering requires both stabilization and measurement of the functional enzyme. Sequence engineering can reduce self-degradation after activation. Active-site titration can determine the functional fraction of the mature enzyme, while time-dependent measurements of protein integrity or residual activity can reveal its loss during use. These measurements distinguish changes in mature-enzyme formation from changes in stability after activation. Zymogenic protease engineering must address both stages to generate active enzymes and maintain their function during catalysis.

## 5. Engineering PTM-Dependent Proteoforms in Fungal Cellulases

Fungal cellulases shift the engineering problem from proteolytic maturation to PTMs and structural architecture. N- and O-glycosylation can alter stability and substrate access, while disulfide formation can constrain flexible regions of the protein. The production host further determines which of these modifications are introduced during folding and secretion. Since these effects depend on where a modification occurs, defined modification sites and the machinery that generates them are more informative engineering targets than the overall extent of glycosylation.

### 5.1. Site-Specific Glycoengineering According to Local Structural Context

*Trichoderma reesei* Cel7A provides a mechanistic basis for site-specific glycoengineering. Amore et al. mapped N- and O-glycosylation and showed that the two glycan classes contribute differently to enzyme function [38]. Removing N-glycans from the catalytic domain had little effect on cellulose conversion or binding but reduced thermal and proteolytic stability. Linker O-glycans influenced cellulose conversion by protecting against proteolysis and were predicted to maintain separation between the catalytic domain and the carbohydrate-binding module (CBM). Qi et al. further examined how the position of N-glycosylation affects Cel7A function by modifying three sites (N45, N270, and N384) [39]. Removal of glycosylation at N384 reduced the thermal transition temperature by approximately 2 °C, while removal at N45 or N384 accelerated activity loss at 62 °C. In contrast, modification of N270 had little effect on thermal stability. Secretion and catalytic activity under standard assay conditions remained largely unchanged. Together, these studies show that the effects of glycosylation depend on both glycan type and position, highlighting the importance of site-specific modifications in glycoengineering.

A recent engineering study provides a direct sequence-level test of this principle. Zuo et al. altered the *Aspergillus terreus* glycoside hydrolase family 12 (GH12) endoglucanase AtEglD by removing its native N65 glycosylation site and introducing a new site near N166 [6]. N65Q eliminated a glycan distant from the catalytic region and increased specific activity on barley β-glucan by 2.73-fold. Catalytic efficiency increased by 74.4%, and activity retention at 60 °C improved. D168S created an N-glycosylation site near N166 and reduced activity toward β-glucan and xyloglucan. Structural analysis placed N166 close to the substrate-binding region and suggested that the added glycan constricted the substrate-binding channel. The mechanism underlying the beneficial effect of removing the more distal N65 glycan was less clear. The Cel7A and AtEglD studies support glycoengineering according to the local structural role of each site rather than a general strategy of increasing or decreasing glycosylation.

The Cel7A and AtEglD studies show that the effects of glycosylation depend on the modified site and the enzyme properties being evaluated. Removing catalytic-domain N-glycans from Cel7A reduced stability but had little effect on cellulose conversion, whereas removing the N65 glycosylation site from AtEglD increased activity toward barley β-glucan. In contrast, introducing an N-glycosylation site near the AtEglD substrate-binding region reduced catalytic activity. These findings are consistent with reports that glycosylation also influences cellulase adsorption to lignin and stability in different expression hosts [10,40]. Therefore, glycoengineering should consider the functional effects of individual glycosylation sites, as overall glycan levels alone may not adequately predict enzyme performance.

### 5.2. Engineering Linker and Structural Architecture

The linker connecting the catalytic domain to the CBM is a functional structural element rather than a passive spacer. A foundational study of *T. reesei* Cel7A showed that glycosylated linkers can interact with cellulose and influence the behavior of the intact multimodular enzyme [41]. Tian et al. treated this architecture as an engineering variable in *T. reesei* cellobiohydrolase II (CBHII) [42]. They shortened the approximately 40-residue linker and varied the number and position of arginine residues. As the deleted segments contained serine and threonine residues, this also reduced the number of predicted O-glycosylation sites. The arginine closest to CBM1 proved essential for efficient folding and expression. Variants CBHII-L-14 and CBHII-L-17, which combined shorter linkers with fewer nonessential arginine residues and fewer potential O-glycosylation sites, increased filter-paper hydrolysis under lignin-rich conditions by 67.7% and 60.5%, respectively, and small-angle X-ray scattering (SAXS) indicated shorter, more rigid solution conformations. These studies show that linker length and charge influence domain organization. Since the same sequence edits can alter PTM potential, both should be considered together in linker design.

The same principle extends from local linker editing to replacement of larger noncatalytic modules. In *Penicillium oxalicum* Cel7A-2, the corresponding linker and CBM1 regions from *T. reesei* Cel7A were transferred separately or together [43]. CBM1 replacement increased Avicel conversion from approximately 22% to 30%. Simultaneous replacement of the linker and CBM1 increased conversion to approximately 40%, indicating an additional contribution from the transferred linker. The transferred linker changed amino-acid composition and glycosylation potential. Glycan occupancy was not measured. This result supports noncatalytic regions as modular engineering elements and shows that linker effects can persist when the catalytic domain is unchanged.

Engineered disulfides can stabilize the protein scaffold without altering the linker or CBM. In *Aspergillus fumigatus* Cel7A, Disulfide by Design 2.0 identified cysteine pairs predicted to form additional disulfide bonds [44]. One of these, T416C/I432C (DSB3) formed a disulfide bond between the A2 and A4 loops and retained approximately 70% activity after 30 min at 70 °C compared with approximately 50% for the wild type. *K*_M_ decreased from 0.128 to 0.081 mM, and *k*_cat_ increased from 4.833 to 9.75 min^−1^. Molecular-dynamics analysis suggested that the crosslink altered local loop geometry and widened the product-exit region from 5.3 to 13.6 Å. Other engineered disulfides reduced stability and activity, indicating that the position matters more than the number of added covalent constraints. Linker editing and disulfide design act through different structural changes, but both require local structural context.

### 5.3. Engineering Host-Dependent Proteoform Generation

Once sequence-level effects are defined, the next question is which proteoforms the production host generates. The production host can determine the final functional state of a cellulase even when the encoded sequence is unchanged. This was demonstrated by expressing the same chimeric cellobiohydrolase (TeTrCBHI) in *S. cerevisiae*, *Yarrowia lipolytica*, and *Lipomyces starkeyi* [5]. The three hosts generated enzymes with distinct molecular and functional properties. Compared with the other forms, the enzyme produced by *Y. lipolytica* was less glycosylated and showed partial degradation in the linker and CBM region. This form also showed lower thermal stability, with a *T*_max_ of 61.8 °C, compared with values close to 65 °C for the enzymes produced by the other hosts. These differences were also reflected in biomass hydrolysis. TeTrCBHI produced in *Y. lipolytica* and *L. starkeyi* converted approximately 70% of the cellulose in pretreated corn stover, whereas the best-performing *S. cerevisiae*-derived form reached approximately 60%. The host affected not only glycosylation but also the catalytic behavior of the resulting enzyme, converting a single cellulase sequence into functionally distinct proteoforms.

If host-dependent processing alters catalyst identity, host pathways themselves become engineering targets. In *Trichoderma atroviride*, Perlińska-Lenart et al. expressed the *S. cerevisiae RER2* gene to increase dolichol supply for glycan assembly [45]. *RER2* expression increased cis-prenyltransferase activity by 93%. O-linked carbohydrate increased by 70%, and N-linked carbohydrate increased by more than fourfold in secreted proteins. Cellulase activity in the culture medium increased twofold. These results suggest that engineering host glycosylation pathways can improve extracellular cellulase activity without modifying enzyme sequences. Unlike the site-specific Cel7A modifications described in Section 5.1, *RER2* expression affects glycan biosynthesis at the host level and may alter multiple secreted proteins. Consequently, the observed increase in activity cannot be attributed to specific proteoforms based on the reported measurements alone. Enzyme-specific glycopeptide analysis combined with activity measurements could help identify the glycosylation changes responsible for this improvement.

Host engineering can also target endoplasmic reticulum (ER) folding and protein-processing capacity. A study in *T. reesei* targeted ER folding and quality-control capacity [46]. The authors overexpressed Pdi1, Ero1, or BiP and manipulated factors involved in ER glycan-dependent quality control. These changes increased β-glucosidase secretion by 29.2–112.5%. BiP overexpression produced the largest increase in total cellulase activity at 149.4% relative to the parental strain. A related study in *P. oxalicum* tested whether strengthening post-translational processing improves functional enzyme quality without a corresponding increase in secretion [47]. HacA was overexpressed to enhance the response to ER folding demand. BipA and PdiA were targeted to support folding and disulfide formation. Enzymatic activity of the homologous amylase Amy15A and heterologous endoglucanase TaEG increased. Amy15A secretion remained unchanged or decreased, and TaEG secretion increased only modestly. The two studies show that engineering ER processing can improve extracellular enzyme production or activity. The *P. oxalicum* results further indicate that higher enzyme activity does not require a proportional increase in protein secretion.

Together, these studies show that the production host influences cellulase performance beyond secretion efficiency. Different production hosts can generate distinct proteoforms from the identical enzyme sequence, while engineering host glycosylation and ER-processing pathways can alter the activity or production of secreted enzymes. Unlike site-specific glycosylation modifications, these approaches affect broader cellular processes and may influence multiple secreted proteins. Measuring enzyme abundance together with glycosylation profiles and folding-state analysis would help determine whether improved activity results from increased enzyme production or more efficient formation of functional proteoforms. These findings extend cellulase engineering from individual protein modifications to host-level control of the final catalyst state.

## 6. Integrating Active-Site Maturation and Oxidative Stability in Lytic Polysaccharide Monooxygenases

LPMOs integrate several limitations that appear separately in the preceding enzyme classes at one reactive copper center. Precise N-terminal processing and copper loading are required to form the active site, whereas PTMs and modular architecture influence its interaction with substrate. Peroxide-driven chemistry can then damage the histidine brace during off-pathway turnover. The following sections examine how LPMO engineering affects active-site formation, substrate engagement, and protection from oxidative damage.

Most fungal LPMOs are synthesized as secreted precursor proteins containing an N-terminal signal peptide rather than a conventional propeptide. Cleavage of this signal peptide generates the mature enzyme and exposes the conserved N-terminal histidine required to form the copper-binding histidine brace.

The mechanistic connection begins at the mononuclear copper center coordinated by the histidine brace. The N-terminal histidine contributes its terminal amino group and imidazole nitrogen, and a second histidine provides the third ligand [48]. Structural analysis of an LPMO-polysaccharide complex showed how the substrate-binding surface positions the polysaccharide relative to this copper center [49]. Later work established H_2_O_2_-driven oxidative cleavage [50]. One-electron reduction generates a Cu(I) state that can support H_2_O_2_-dependent substrate oxidation, but poor substrate positioning exposes the same copper center to damaging peroxide chemistry [11,51]. Together, these studies provide the mechanistic basis for the engineering examples below. The reactive copper chemistry that supports catalysis also creates a need for precise maturation and redox protection.

### 6.1. Engineering Processing-Dependent Active-Site Formation

Signal-peptide cleavage plays a direct role in formation of the LPMO active site, which distinguishes it from the protease maturation described above. In zymogenic proteases, prodomain removal activates a catalytic domain that is already encoded within the precursor. In LPMOs, cleavage of the signal peptide generates the mature N terminus and exposes the free α-amino group of the conserved histidine required for copper coordination. This requirement can become a bottleneck during heterologous secretion if processing leaves additional residues ahead of the native histidine. To address this problem, a recent study compared secretion architectures in *P. pastoris* to identify a system supporting both efficient secretion and correct N-terminal processing of fungal LPMOs [13]. The yeast-derived OST1 signal peptide supported both, producing catalytically active LPMOs, whereas the pre-OST1-pro-α-factor architecture left an EAEA extension on the N-terminus, leading to loss of activity. Mass analysis confirmed the correctly processed N-terminal peptide HTIFQK in the active enzyme, whereas the incorrectly processed form retained the sequence EAEAHTIFQK. These results show that secretion alone is insufficient for LPMO maturation. The cleavage boundary must generate the native N-terminal histidine required to form the copper-binding active site.

The N-terminal H1 is also subject to histidine methylation in fungal LPMOs, introducing an additional maturation step at the same residue required for copper coordination. Batth et al. sought to identify the responsible enzyme and to determine whether correct formation of the mature N terminus is required for methylation [12]. Quantitative proteomics combined with CRISPR/Cas9 knockout screening in *Aspergillus nidulans* identified *nhmT* (AN4663) as the N-terminal histidine methyltransferase (NHMT). The authors then co-expressed NHMT with LPMO LsAA9A in *K. phaffii* and compared secretion signals differing in their ability to expose the native H1. The native LsAA9A and *A. niger* α-amylase signal peptides generated the correct N terminus and supported H1 methylation. In contrast, the *S. cerevisiae* α-mating-factor leader left an additional residue before H1, and methylation was not detected. These results extend the importance of N-terminal processing beyond copper-site formation by showing that precise exposure of H1 is also required for its subsequent methylation.

These maturation steps can influence later catalytic stability. Direct comparison of methylated and unmethylated TaLPMO9A showed small effects on substrate preference and copper affinity [52]. The redox potential changed little. H1 methylation increased resistance to oxidative self-inactivation and improved performance under conditions that promote reactive-oxygen chemistry. The later NHMT study associated H1 methylation with protection against auto-oxidative inactivation [12]. A quantum mechanics/molecular mechanics (QM/MM) analysis tested possible oxidation pathways of the copper-coordinating histidines [53]. The calculations identified a [CuO]^+^ intermediate capable of abstracting hydrogen from N-terminal H1 and initiating oxidative damage. H1 methylation increased the calculated activation barrier by approximately 10 kJ/mol. Substrate binding raised the barrier further. The experimental and computational results show that correct processing enables H1 methylation and that this modification can extend the lifetime of the copper-binding active site.

### 6.2. Tuning Copper Reactivity Through the Second Coordination Sphere

Copper loading establishes the LPMO metal center, but the surrounding protein environment controls its reactivity. Hall et al. tested the conserved second-sphere residue Q164 in the fungal AA9 LPMO NcAA9C [54]. Replacing it with Glu, Asp, or Asn produced little change in the resting Cu(II) coordination environment but changed redox properties and reaction kinetics. Negatively charged substitutions lowered the reduction potential, with Q164E showing the strongest effect. This variant showed a 5-fold increase in oxidase activity and a higher initial rate of cellopentaose conversion under reductant-driven conditions, but was also more susceptible to oxidative inactivation. Computational analysis suggested altered peroxide reactivity and O–O bond cleavage. A related study in the bacterial AA10 LPMO MaAA10B showed that Q219E increased oxidase activity and shifted catalytic preference toward chitin [55]. Substitutions within the surrounding HQY motif produced distinct changes in catalytic performance. These studies show that second-sphere residues can influence copper reactivity without changing the primary copper ligands. However, increased oxidase activity does not indicate more productive polysaccharide oxidation, particularly when improved initial activity is accompanied by faster oxidative inactivation. Therefore, evaluating these variants requires measurements of substrate conversion and activity loss under defined reaction conditions, including reductant and peroxide availability. These measurements can help distinguish improved catalytic performance from increased copper reactivity that also promotes enzyme inactivation.

Interpreting these effects requires careful control of copper that is not bound to the enzyme. In a bacterial AA10 LPMO, purified enzyme was saturated with Cu(II), excess copper was removed, and the preparation was verified to contain stoichiometric enzyme-bound copper with negligible free metal before reductant-dependent reactions were compared [56]. Micromolar free copper increased H_2_O_2_ formation in the presence of ascorbate and raised apparent cellulose oxidation, whereas gallic-acid-driven reactions were less sensitive to free copper. This study shows that free copper can alter H_2_O_2_ supply through reductant oxidation and affect measured LPMO activity. Apparent increases in LPMO reactivity should not be attributed to changes in enzyme-bound copper unless free-copper effects have been excluded.

The redox behavior of enzyme-bound copper also changes during catalysis. A study of NcLPMO9C tested whether repeated H_2_O_2_-driven turnover requires continuous reduction in the copper center and how substrate binding influences its redox state [51]. The authors followed copper-state changes during peroxide-driven reactions and compared the enzyme in the presence and absence of xyloglucan. A single priming electron supported up to 20 catalytic cycles, while the copper center remained predominantly in the reduced Cu(I) state after turnover. Xyloglucan binding increased the redox potential of the copper site and favored retention of this reduced state. In the absence of substrate, transient species associated with protective radical chemistry became more apparent. These observations connect copper redox behavior to substrate engagement and provide a mechanistic basis for engineering the protein environment around the copper center. The surrounding protein must support productive oxidation while limiting damage caused by peroxide chemistry.

### 6.3. Engineering Modular Architecture for Productive Substrate Engagement

Substrate engagement is also shaped by the modular architecture surrounding the LPMO catalytic domain. Many LPMOs carry a CBM connected to the catalytic domain through a flexible linker, but the functional contribution of the linker has remained less clear than that of the CBM. A study of *Botrytis cinerea* BcAA9C tested whether the linker functions only as a spacer or contributes directly to substrate binding and catalytic performance [57]. The authors generated variants with different linkers and compared them with the full-length enzyme and a construct lacking the CBM. Removing the CBM decreased oxidation of polymeric cellulose by approximately 93%, whereas linker truncation reduced activity by 34–79% across the constructs. Removal of the native linker also reduced binding to the polymeric substrate by 7-fold. The full-length linker also improved thermal stability, particularly at elevated temperatures. These effects occurred even though linker modification had little effect on oxidation of the soluble Amplex Red substrate, indicating that the catalytic center itself remained functional. The results show that the linker contributes to productive engagement with polymeric substrate and helps maintain the functional architecture of the modular enzyme. This makes the linker a potential engineering target rather than a passive connection between the catalytic domain and CBM.

Linker architecture was examined more systematically in the two-domain LPMO ScLPMO10C, with the aim of determining how linker length and sequence composition influence the conformation, activity, and stability of the intact enzyme [58]. To separate linker length and sequence composition, the authors compared variants carrying the same linker sequence at different lengths with a variant containing a different linker sequence of similar length to the wild type. The wild-type and serine-rich variants outperformed both the shorter and longer variants, indicating that linker length is a major determinant of productive domain organization. Interestingly, the serine-rich variant also showed substantially higher thermostability despite a linker length close to that of the wild type, showing that sequence composition further modulates stability. The intact two-domain protein also unfolded cooperatively, whereas the isolated catalytic domain and CBM unfolded independently. The linker therefore organizes the catalytic domain and CBM as an integrated functional unit, providing a basis for engineering both linker length and sequence composition in modular LPMOs.

Whereas linker engineering modifies an existing modular LPMO, domain fusion can create a new substrate-binding architecture. An earlier mutational analysis of SmLPMO10A showed that changes on the substrate-binding surface affected both overall performance and protection from off-pathway chemistry without altering the catalytic copper site [59]. Gao et al. applied this principle by adding the CBM of MtLPMO9G to the naturally single-domain MtLPMO9L [18]. The fusion showed stronger substrate binding, higher catalytic activity, and greater H_2_O_2_ tolerance. Molecular-dynamics simulations indicated that the CBM held the catalytic domain closer to the polysaccharide surface. The engineering effect therefore extended beyond adsorption. Increasing the fraction of enzyme positioned at substrate promoted productive H_2_O_2_ use and reduced off-pathway self-oxidation.

The role of the CBM can extend to regulation of copper chemistry beyond substrate binding. In 2025, Forsberg et al. demonstrated that a conserved MMH motif in CBM2 domains contributes to the redox stability of cellulose-active LPMOs through a preference for Cu(I) [60]. Sequence and structural analyses identified the motif specifically in CBM2s associated with AA10 LPMOs. The authors then removed the MMH motif from ScLPMO10C and introduced it into the closely related mgLPMO10, which naturally lacks this motif. The MMH-containing CBM2s preferentially bound Cu(I), while these modifications had little effect on cellulose binding. The motif also influenced the reactivity of the catalytic copper and reduced damaging redox chemistry under substrate-free conditions. The study supports two protective roles for the CBM2. It can capture free copper released into solution and can interact with the reduced catalytic copper through the MMH site. This finding represents an engineering strategy that is not captured by the conventional function of the CBM as a carbohydrate-binding module.

Natural multidomain LPMOs provide additional examples of how CBMs can regulate catalytic function. A recent study characterized the tetra-modular EcLPMO from *Enterobacter cloacae* to determine the roles of its two domains of unknown function (DUFs) and C-terminal CBM73 [61]. The authors compared the full-length protein with domain-truncated variants to determine how each accessory region contributes to chitin degradation. CBM73 strongly enhanced chitin binding and promoted synergy with the endogenous chitinase EcChiA, whereas deletion of the two DUF domains reduced LPMO activity despite a negligible effect on substrate binding. Structural modeling provided a possible explanation for this effect. DUF-B contains two conserved histidines that were predicted to coordinate the copper center together with two histidines from the AA10 domain, forming an interdomain tetra-histidine coordination site. This arrangement was proposed to regulate the catalytic copper center and potentially protect it from oxidative damage. It reveals interdomain metal coordination as a potential design principle for controlling copper reactivity in modular LPMOs.

### 6.4. Engineering Redox Protection and Catalytic Lifetime

The previous section showed how substrate engagement improves LPMO performance. A structural study of chitin-active SmLPMO10A tested whether substrate binding also protects the copper center from oxidative damage [11]. Damage appeared first around the copper center and was followed by broader structural perturbations and loss of catalytic activity, whereas pre-incubation with chitin reduced these changes and preserved activity. Productive substrate engagement therefore directs reactive chemistry toward polysaccharide oxidation and away from self-damage.

LPMOs can also protect the copper center by transferring oxidizing equivalents away from the histidine brace. In 2023, Zhao et al. tested whether residues surrounding the metal center can redistribute the oxidizing equivalent and limit self-inactivation [62]. Generating peroxide-derived intermediates, they identified an initial high-valent state consistent with a Cu(II)-histidyl radical. They combined mutational analysis with spectroscopic characterization and time-dependent density functional theory (TD-DFT) calculations to follow the fate of this radical. The results supported transfer of the oxidizing equivalent from the histidine brace to a nearby tyrosine (Y164), generating a Cu(II)-tyrosyl radical while returning the copper-coordinating histidine toward its resting state. Disrupting this pathway with the Y164F substitution increased oxidative damage and accelerated enzyme deactivation. These results show that residues surrounding the copper site can form a protective radical-transfer pathway that limits damage to the histidine brace during peroxide-driven catalysis.

A subsequent study extended this local protection mechanism to a longer hole-hopping pathway [63]. Sequence analysis of AA10 LPMOs identified a conserved network of aromatic residues (e.g., W119, W178) extending from the active-site region toward the protein surface. Guided by this analysis, the authors mutated individual positions in SmAA10A and followed their effects on radical formation and enzyme stability. W178, located closest to the copper center within this network, was required for formation of the characteristic protein radicals and for protection against oxidative inactivation. W119 was identified as a subsequent position in the transfer pathway, while the surface-exposed Y121–H160 region contributed to dissipation of the oxidizing equivalent. Mutations in this pathway also revealed a trade-off between catalytic activity and oxidative stability. The W119F variant showed higher initial peroxygenase activity but inactivated more rapidly under elevated H_2_O_2_ supply. Slower hole transfer retained more oxidizing equivalents for productive catalysis while reducing the capacity of the protein to dissipate damaging redox equivalents. The NcAA9C Q164E variant described in Section 6.2 showed a similar pattern, with increased oxidase activity accompanied by faster oxidative inactivation [54]. Although these variants were examined in different LPMO systems using different activity measurements, both illustrate that higher initial activity does not necessarily lead to sustained catalytic performance. Therefore, evaluating product formation and residual enzyme activity over time under controlled substrate and peroxide conditions can help identify variants that maintain productive oxidation during turnover.

Stabilizing the protein scaffold provides another way to improve LPMO stability without directly modifying redox-protection pathways. In CjLPMO10A, an engineered N78C/H116C disulfide increased apparent *T*_m_ by approximately 7 °C, extended the half-life at 60 °C 3-fold, and increased specific activity about 1.5-fold while retaining catalytic function [64]. A second study applied computer-aided stabilization to MtLPMO9V [65]. The M3 variant combined an A170C/A175C disulfide with Q120Y, increased activity by 88%, and increased catalytic efficiency by 1.90-fold. M3 showed improved thermostability and retained synergy with the endoglucanase DtCelA. These studies support scaffold stabilization as a means of improving thermal tolerance, although they do not directly establish longer operational lifetimes under sustained catalysis or reduced oxidative self-damage.

These studies illustrate different strategies for improving LPMO performance and resistance to inactivation. Improved substrate engagement can favor productive polysaccharide oxidation, while radical-transfer pathways help protect the histidine brace from oxidative damage. Scaffold stabilization through disulfide engineering may also help preserve catalytic activity by improving protein stability. Because these strategies address different mechanisms of activity loss, their effects cannot be evaluated from initial activity or thermal stability alone. Measuring product formation and residual activity over time provides a more direct assessment of catalytic persistence, while examining damage to the copper center can help explain the underlying inactivation mechanism.

### 6.5. LPMOs as an Integrated Model of Catalyst Maturation and Oxidative Stability

The LPMO examples define a connected sequence of events that determines whether an expressed protein becomes a productive and persistent catalyst. Correct signal-peptide processing first generates the native N-terminal H1 required for copper coordination. Copper incorporation then completes the catalytic metal center, while the surrounding protein architecture determines how effectively the enzyme engages polysaccharide substrate. Once catalysis begins, local redox-protection pathways and overall structural stability govern how long the active state is maintained. These steps can be examined separately, but a change introduced at one stage can alter the functional state carried into the next.

This dependence between stages explains why improvement of a single property does not necessarily increase overall catalytic output. Incorrect N-terminal processing prevents formation of the native copper-binding site, while changes around the metal center can alter peroxide reactivity and increase susceptibility to oxidative damage. An active LPMO can also lose function rapidly when substrate engagement is insufficient to keep reactive chemistry coupled to productive oxidation. Engineering the modular architecture can address several of these constraints at once. For example, adding a CBM to MtLPMO9L increased substrate binding and promoted more productive H_2_O_2_ use, improving catalytic performance while reducing oxidative inactivation [18]. Effective LPMO engineering must therefore coordinate active-site formation, substrate binding, and catalyst lifetime throughout the catalytic cycle.

LPMOs serve as a useful integrated case because maturation, substrate engagement, and oxidative stability can all be linked to measurements at the same catalytic center. This combination provides a transition from engineering the mature catalytic sequence toward strategies that target the cellular and structural processes that generate and maintain functional enzyme states.

## 7. Generalizing Engineering Strategies for Catalyst Formation and Maintenance

The four enzyme classes reveal recurring engineering strategies rather than four unrelated collections of examples. In P450s, cofactor loading controls holoenzyme formation and redox-system organization controls productive turnover. In zymogenic proteases, precursor folding and host processing govern formation of the mature enzyme, while preservation of the mature state determines how long it remains functional during catalysis. Fungal cellulases show how site-specific PTMs and noncatalytic architecture shape the proteoform generated by the production host. LPMOs bring these problems together at one reactive copper center, where maturation influences subsequent substrate engagement and catalytic lifetime. Across these systems, enzyme engineering can target the processes that create and maintain the functional catalyst in addition to the mature catalytic sequence. Table 2 summarizes these findings by showing how engineering strategies address different limitations in enzyme function and how their effects have been evaluated.

Comparing these enzyme classes also reveals trade-offs associated with different engineering strategies. In P450 production, Kato et al. found that increasing intracellular 5-ALA supply could reduce the expression of larger hemoproteins, requiring sequential induction to balance cofactor supply and target-protein production [23]. In Cel7A, removing catalytic-domain N-glycans had little effect on cellulose conversion but reduced thermal and proteolytic stability [38], whereas modifying glycosylation sites in AtEglD increased or decreased activity depending on their position [6]. In computationally designed proenzymes, stronger suppression of catalytic activity before cleavage was accompanied by lower activity recovery after proteolytic activation [66]. LPMO variants showed a similar trade-off, as increased initial oxidase or peroxygenase activity could be accompanied by faster oxidative inactivation [54,63]. These findings show that improving one property does not necessarily improve overall enzyme performance. An important remaining question is whether improvements in functional-enzyme formation or initial activity lead to greater cumulative product yields during prolonged catalysis. Therefore, engineering strategies should address the limiting step and be evaluated for their effects on catalyst formation and catalytic persistence under the intended reaction conditions.

These principles extend beyond these four enzyme classes. Maturation machinery can be engineered when activation or cofactor assembly limits catalyst formation. Inactive-to-active transitions can be introduced as designed properties. Productive conformational states can be enriched or stabilized. Computational methods can treat state-resolved biochemical or structural measurements as design information. The following sections generalize these strategies by the molecular transition being engineered rather than by enzyme family.

### 7.1. Engineering Catalyst-Forming Machinery

The P450, protease, and cellulase examples show that cellular processes outside the mature catalytic sequence can limit functional-enzyme formation. P450s require heme incorporation. Proteases require signal-peptide or propeptide processing. Cellulases require host pathways that support glycosylation and ER folding. Each example shows the same principle. Increasing catalytic-protein expression alone may not increase the active fraction when a maturation step is limiting. Similar limitations occur in enzymes that require a separate activation reaction or dedicated cofactor-assembly machinery.

Carboxylic acid reductases (CAR) provide an example in which the catalytic protein must undergo post-translational activation before turnover can occur. Their carrier-protein domain requires phosphopantetheinylation by a phosphopantetheinyl transferase. Choi et al. examined whether transferase capacity limits CAR-dependent whole-cell catalysis and increased the copy number of the phosphopantetheinyl transferase gene *sfp* without changing the CAR sequence [67]. In *E. coli* expressing the *Mycobacterium smegmatis* CAR MSM5586, tandem expression of three *sfp* copies increased 1,4-pentanediol production by 1.43-fold relative to the single-copy system. The same strategy improved product formation when applied to a *Mycobacterium abscessus* CAR. The study identifies phosphopantetheinyl transferase capacity as an engineering target.

[NiFe] hydrogenase separates soluble protein production from active catalyst formation. Fan et al. first established high-level production of the *Cupriavidus necator* regulatory hydrogenase in *E. coli*, yielding approximately 280 mg/L soluble protein [68]. However, the purified enzyme was inactive because the large subunit lacked the [NiFe] catalytic cofactor, even though Fe–S clusters had formed in the small subunit [69]. A subsequent study introduced the *C. necator* hydrogenase maturation machinery into *E. coli* to restore active-site assembly [69]. Co-expression of the Hyp maturation proteins together with improved nickel uptake through the HoxN nickel transporter enabled incorporation of the [NiFe] center and production of catalytically active hydrogenase. The resulting production system was later transferred to high-cell-density fed-batch cultivation, yielding more than 130 mg/L active enzyme [70]. These studies separate high-level protein production from cofactor-dependent formation of the active catalyst and show that maturation capacity can become an independent engineering target.

The same principle can be extended beyond native maturation systems. A recent study generated an artificial enzyme containing a non-natural catalytic group [71]. The authors used a synthetic secondary-amine organocatalytic cofactor, termed cofactor_SA, together with the LmrR protein scaffold. An engineered cysteine at position 57 allowed the cofactor to be covalently anchored within the protein pocket through disulfide-bond formation after cellular uptake. This intracellular assembly generated a secondary-amine catalytic site capable of supporting the Mannich reaction. Formation of the assembled catalyst was confirmed before the surrounding protein sequence was optimized. Directed evolution was then used to improve the catalytic environment around the installed cofactor. The resulting artificial Mannichase catalyzed highly stereoselective reactions in whole cells. This study extends catalyst-formation engineering from improving native maturation pathways to designing the intracellular assembly process required to generate a new functional catalyst.

These examples generalize the maturation problems identified in P450s and cellulases to a broader engineering principle. When catalytic competence requires an activation reaction or cofactor-assembly step, the capacity of that process can determine how much expressed protein becomes functional enzyme. Engineering the catalytic sequence alone may not address this limitation.

### 7.2. Engineering Controlled Transitions Between Inactive and Active States

The protease examples show that maturation can determine when a catalytic domain becomes active. The same logic has been introduced into enzymes that do not naturally exist as zymogens. An early example used alternate-frame folding to convert barnase into an artificial zymogen activated by HIV-1 protease [72]. The proenzyme showed a 250-fold suppression of *k*_cat_/*K*_M_ relative to wild-type barnase. Proteolytic cleavage increased *k*_cat_/*K*_M_ by 130-fold. This work established the inactive-to-active transition itself as an enzyme design property rather than only a feature inherited from a natural precursor.

Carboxypeptidase G2 (CPG2) provides a more recent implementation because the native enzyme is not produced as a zymogen. A Rosetta-based study introduced a protease-responsive inactive state using a circularly permuted CPG2 scaffold with new termini near the active site [66]. Inhibitory prodomains were attached through protease-cleavable linkers. Selected designs reduced catalytic efficiency by up to 80% before cleavage and recovered activity after proteolysis. Interdomain disulfide bonds strengthened the inactive state, increasing inhibition to 98%. The more constrained variants recovered less activity after cleavage. The barnase and CPG2 studies define a common design trade-off. The pre-activation state must suppress catalysis, yet cleavage must still recover a productive state.

A complementary strategy applies where an enzyme is naturally produced as an inactive precursor but its conversion to the mature form limits production. Protein-glutaminase is synthesized as a proenzyme that becomes active only after proteolytic removal of its propeptide. Teng et al. tested whether precursor production and subsequent proteolytic activation could be combined in *B. subtilis* [73]. After identifying AprE as an activating protease, the authors co-expressed pro-protein-glutaminase with AprE, which increased the conversion of the precursor to mature enzyme. The optimized self-activating system reached 53.0 U/mL mature protein-glutaminase activity in a 5 L bioreactor. This study shows that production of a functional enzyme can be improved by engineering the precursor-to-mature conversion step itself.

These studies generalize the maturation logic observed in zymogenic proteases. One route introduces an inactive precursor state into an enzyme that does not naturally contain one. The other improves a native activation step by coordinating precursor production with its processing machinery. Both strategies treat activation as a controllable transition. The design problem is to generate enough protection before activation while preserving efficient conversion to the functional catalyst.

### 7.3. Engineering Productive Molecular States

The P450 and LPMO examples show that catalyst formation does not define a single functional state. In P450s, productive conformations position redox partners for electron transfer. In LPMOs, substrate positioning and local redox chemistry determine productive turnover. Engineering can therefore target the conformational states that support catalysis. Otten et al. provided experimental evidence that directed evolution can recover catalytically important dynamics [16]. Starting from an impaired cyclophilin A variant, two second-shell mutations partly restored catalysis. Nuclear magnetic resonance (NMR) and room-temperature crystallography linked the improvement to faster interconversion between two catalytically essential conformational substates. A recent lactate-dehydrogenase study offers another sequence-level example [74]. Q86R and I227V altered oxaloacetate recognition and the conformational behavior of an active-site loop, and the combined variant produced the highest catalytic efficiency toward oxaloacetate among the tested variants. Formation of a closed state alone did not define a productive configuration because substrate positioning within that state remained important. These studies show that engineering productive conformations involves more than stabilizing a single protein structure.

Conformational landscapes can also be engineered computationally. St-Jacques et al. used multistate computational design to remodel the open-closed equilibrium of aspartate aminotransferase [75]. The design stabilized a less populated but reactive conformation and increased catalytic efficiency toward a non-native substrate by up to approximately 100-fold. Selectivity switches reached up to 1900-fold. Room-temperature crystallography and multitemperature NMR confirmed the intended shift in conformational equilibrium. A Baeyer–Villiger monooxygenase study further showed that even adjacent mutations can produce different changes in catalytic properties [76]. The neighboring Y141L and Y142L substitutions produced different changes in catalytic efficiency and productivity. They also differed in regioselectivity and conformational mobility. These studies show that sequence changes can alter the conformational states available to an enzyme. Computational design can target those states before experimental screening, while analysis of conformational behavior can help explain differences in catalytic performance.

Screening should match the molecular property a given strategy targets. Initial activity identifies variants with rapid turnover but provides limited information about catalyst lifetime. Coupling efficiency is needed when reducing equivalents can enter unproductive pathways. Time-dependent activity or active-site measurements show how quickly catalytic competence is lost. Conformational measurements distinguish variants that populate different catalytic states despite similar static structures. The conformational studies show how sequence changes can improve productive turnover, whereas the P450 and LPMO examples show that catalytic lifetime requires separate measurements. Since a single early activity measurement cannot distinguish these effects, multi-objective evaluation is necessary wherever the engineering goal is total catalytic output.

### 7.4. Data-Guided Design for Functional-State Engineering

The strategies above create a measurement problem for computational enzyme design. Current sequence and structure models can search large design spaces, but catalyst formation and catalytic persistence are measured less often than endpoint activity. Conformational populations are also measured less often. A recent review of generative enzyme design identified limited representation of dynamics and physicochemical constraints as a major gap in current workflows [77]. The multistate redesign of aspartate aminotransferase shows the value of representing alternative functional conformations [75]. The maturation and lifetime problems discussed here call for a similar shift toward experimentally resolved catalyst states rather than a single endpoint activity label.

The four enzyme classes provide examples of measurements that could be retained as design information. P450 can report holoenzyme fraction together with coupling efficiency. Protease studies can distinguish precursor abundance from the concentration of mature active enzymes. Cellulase studies can record the production host and the proteoform generated under those conditions. LPMO studies can connect processing state with copper-site measurements and catalytic lifetime. These measurements define experimentally resolved states that can be linked to sequence changes and used as design targets instead of treating final activity as the only label.

Recent advances in structure prediction and protein design, including AlphaFold 3, ProteinMPNN, LigandMPNN, and RFdiffusion2, have expanded the use of structural information in enzyme engineering (Table 3) [78,79,80,81]. However, a predicted protein structure alone does not establish whether the designed enzyme can form and maintain a catalytically competent state. Recent metalloenzyme studies illustrate how computational design can address catalytic-site organization and the stability of functional conformations. Kim et al. combined quantum-chemically defined catalytic geometries with RFdiffusion2 to construct zinc-dependent metallohydrolases [82]. The designed proteins showed zinc-dependent hydrolytic activity, and the crystal structure of an active design closely matched the predicted catalytic architecture. Wagner Egea et al. examined a different challenge in a de novo metalloenzyme with lanthanide-dependent photoenzymatic activity [83]. Structural and spectroscopic analyses identified an alternative conformation that limited productive metal binding. The authors used ProteinMPNN to redesign the sequence and stabilize the active conformation, accelerating metal binding and increasing catalytic efficiency by approximately 10-fold. King et al. also applied ProteinMPNN to a non-heme Fe(II)/α-ketoglutarate enzyme, improving its stability and expression while retaining catalytic function [84]. The stabilized enzyme provided a better starting point for subsequent directed evolution of a non-native reaction. These studies show that computational design can address both catalytic-site organization and the structural features needed to maintain enzyme function.

**Figure 3:**
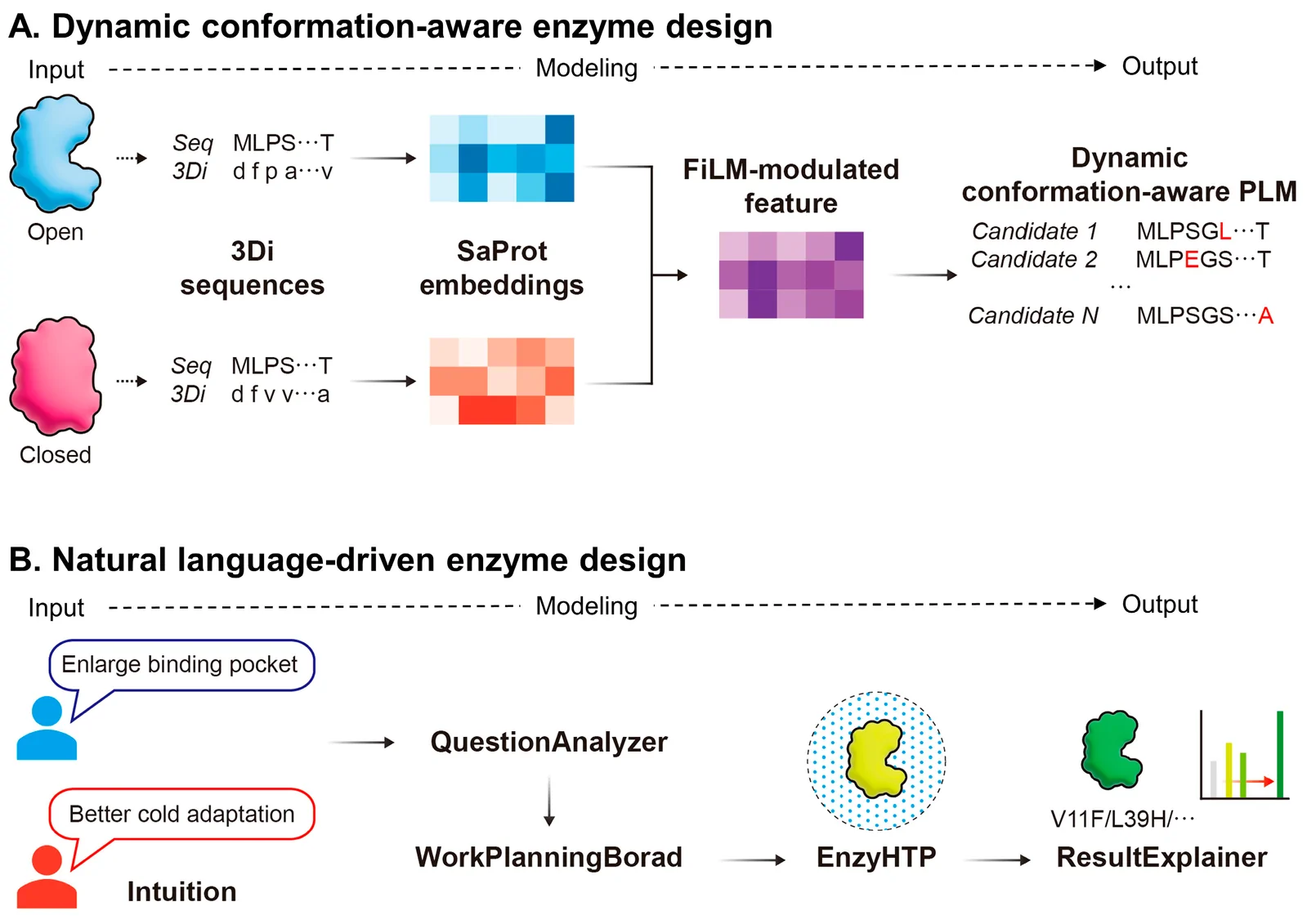
Representative computational workflows for functional-state enzyme engineering. (**A**) The dynamic conformation-aware protein language model (DC-PLM) uses open and closed conformations as structural inputs. Each conformation is encoded as a 3Di sequence and combined with the amino-acid sequence to generate state-specific SaProt embeddings. Differences between the two states are incorporated through feature-wise linear modulation (FiLM) to prioritize candidate mutants. (**B**) Natural language-driven enzyme design converts an intuition-based engineering request into a physics-based computational workflow. QuestionAnalyzer refines the engineering objective, and WorkPlanningBoard defines computable metrics and mutation plans. EnzyHTP performs the simulations, and then ResultExplainer interprets the results and prioritizes candidate mutants.

Conformational information can also be incorporated directly into variant design. A study of *Candida antarctica* lipase B tested whether representing multiple catalytic conformations (i.e., open and closed lid states) could improve sequence selection beyond a single static structure (Figure 3A) [85]. The authors encoded both structures using the 3D interaction (3Di) structural alphabet, which represents local three-dimensional environments as sequence-like states, and used the structure-aware protein language model SaProt to generate representations of each conformation for variant selection. This strategy allowed sequence changes to be evaluated by their predicted effects on the relationship between the two states. Five rounds of model-guided design and experimental testing produced the M5 variant, which showed an 8.6-fold increase in *V*_max_ and a 16.5-fold increase in *k*_cat_ relative to the wild-type enzyme. Molecular-dynamics simulations indicated that the selected substitutions favored lid opening and supported productive substrate positioning. This study shows that conformational states can serve as design inputs before experimental screening, allowing dynamic features of the functional enzyme to contribute directly to sequence selection.

A complementary strategy converts a mechanistic engineering hypothesis into quantitative molecular properties before variant selection. MutexaGPT was developed for this purpose by linking language-model agents with the EnzyHTP molecular-modeling platform (Figure 3B) [86]. The system translates a user-defined engineering objective into a simulation workflow and uses the calculated physical properties to prioritize mutations. In halide methyltransferase engineering, the authors examined whether enlarging the substrate-binding cavity could improve acceptance of bulkier substrates. MutexaGPT used cavity properties derived from molecular simulations to prioritize mutations within a focused candidate library. The top-ranked candidates included four previously validated hits, corresponding to a 40% hit rate and approximately 4-fold enrichment over the baseline strategy. The same framework was applied to a bidomain amylase, where linker dynamics were evaluated to identify variants with improved activity at low temperature. This approach does not directly model maturation events such as cofactor loading or host-dependent processing, but it shows how mechanistic hypotheses about functional enzyme states can be converted into quantitative descriptors that guide sequence selection before experimental screening.

These approaches provide a route for extending data-guided enzyme engineering beyond endpoint activity by allowing candidate mutations to be evaluated according to their predicted effects on specific functional states. Multi-state structural models can represent changes in conformational behavior, while physics-based calculations can examine local interactions and cofactor coordination. Experimental measurements are then needed to determine whether the predicted changes improve functional-enzyme formation, productive catalysis, or catalytic persistence. The appropriate measurements depend on the targeted process. Holoenzyme fraction and precursor-to-mature conversion can assess catalyst formation, while proteoform characterization and active-site concentration provide further information on the molecular state of the enzyme. Coupling efficiency can evaluate productive turnover, and time-dependent activity measurements can reveal how well catalytic function is maintained. Linking these measurements to protein sequence and production context would help guide subsequent rounds of engineering and support models that account for the formation and maintenance of functional enzymes.

## 8. Conclusions

The studies reviewed here support an expanded view of enzyme engineering in which sequence optimization is considered alongside the processes that generate and maintain the functional catalyst. P450s highlight cofactor incorporation and productive redox organization. Proteases show how precursor folding and processing control formation of the mature enzyme. They also show how autolysis limits persistence after activation. Fungal cellulases show the contribution of site-specific PTMs and noncatalytic architecture. They further show how host-dependent processing changes the final proteoform. LPMOs connect active-site maturation with substrate engagement and preservation of a reactive catalytic center.

Two questions provide a common framework across these systems. How much expressed protein reaches a catalytically competent state? How long does that state remain productive during turnover? Catalyst-forming machinery determines formation of the active molecular state. Protein architecture and conformational organization control productive turnover, and protective mechanisms control catalytic lifetime. Sequence changes can influence each of these levels, but the limiting step may lie outside the mature catalytic sequence.

This framework also changes how engineering outcomes should be evaluated. Expression level alone cannot establish how much active catalyst forms. Initial activity does not reveal how long that catalyst remains functional. Measurements should resolve the molecular state a given strategy targets. Holoenzyme fraction reports cofactor loading. Precursor-to-mature conversion reports processing. Proteoform identity and active-site concentration address the functional state of the enzyme population. Coupling efficiency and time-dependent activity report productive turnover and persistence. These state-resolved data can be incorporated into computational design and multi-objective screening.

Sequence engineering remains central to biocatalyst development, but its effects become more informative when interpreted through the processes that convert sequence into a functional catalyst and preserve that catalyst during use. Grouping studies by recurring mechanistic strategies shows where different interventions address the same underlying problem and where further measurements are needed to distinguish catalyst formation from catalyst maintenance.

## Figures and Tables

**Table 1:** Key measurements for evaluating catalyst formation, productive catalysis, and catalytic lifetime.

Engineering Stage	Key Measurements	What It Shows	Representative System	Ref.
Catalyst formation	Holoenzyme fraction, precursor:mature ratio, proteoform analysis, active-site titration	Extent and molecular state of catalyst formation	P450, protease, cellulase	[2,5,14,15]
Productive catalysis	Coupling efficiency, substrate-binding analysis, conformational-state analysis	Efficiency and organization of productive turnover	P450, LPMO, conformationally dynamic enzymes	[16,17,18]
Catalytic lifetime	Time-dependent residual activity, structural integrity	Persistence of catalytic competence during turnover	Protease, LPMO	[9,11]

**Table 2:** Functional bottlenecks, engineering strategies, and supporting evidence across the four enzyme classes.

Enzyme Class	Limiting Step	Engineering Approach	Supporting Evidence	Main Limitation	Ref.
Cytochrome P450	Heme incorporation, productive redox coupling	Heme supply, redox-partner and linker engineering	Holoenzyme fraction, coupling efficiency, activity retention	Expression or product yield alone cannot establish coupling	[2,17,23,31,32]
Zymogenic protease	Precursor processing, mature-enzyme loss	Prodomain and cleavage-site redesign, host processing, autolysis reduction	Precursor:mature ratio, active-site titration, activity retention	Extracellular activity does not isolate maturation from turnover	[9,14,33,37]
Fungal cellulase	Host-dependent PTMs, substrate access	Site-specific glycosylation, linker and secretion-pathway engineering	Site occupancy, proteoform analysis, substrate conversion	Glycan effects depend on position, host and substrate	[6,38,39,45]
LPMO	Copper-site maturation, oxidative inactivation	N-terminal processing, copper-site and redox protection, CBM engineering	Correct N terminus, copper state, product formation, time-dependent loss	High initial oxidation can accompany faster inactivation	[13,52,54,63]

**Table 3:** Representative computational approaches for functional-state enzyme engineering.

Application	Tool or Approach	Main Input	Role in Enzyme Design	Key Limitation	Ref.
Structure and complex modeling	AlphaFold 3	Protein sequence + ligand/ion/modified residue context	Models structures containing cofactors, ligands, or modified residues	Does not directly model catalytic dynamics or maturation pathways	[78]
Backbone-guided sequence design	ProteinMPNN	Protein backbone + fixed-residue constraints	Designs sequences compatible with a specified backbone and supports scaffold stabilization	Does not explicitly model catalytic chemistry or ligand interactions	[81,83,84]
Context-aware sequence design	LigandMPNN	Protein backbone + nonprotein atomic context	Designs sequences around ligands, cofactors, or metals	Requires a predefined structural context	[79]
De novo active-site design	RFdiffusion2	Catalytic functional-group geometry	Generates protein scaffolds around catalytic groups	Focuses on active-site scaffolding rather than maturation or catalytic lifetime	[80,82]
State-aware variant design	DC-PLM	Amino-acid sequence + open/closed 3Di representations	Selects variants using alternative conformations	Depends on representative conformational states	[85]
Intuition-guided variant design	MutexaGPT	Natural-language engineering objective	Converts mechanistic intuition into physical descriptors and ranked variants	Limited to implemented physical metrics and simulation workflows	[86]

## Data Availability

No new data were created or analyzed in this study. Data sharing is not applicable to this article.

## Abbreviations

The following abbreviations are used in this manuscript:

CAR	carboxylic acid reductase
CBM	carbohydrate-binding module
CBH	cellobiohydrolase
CPR	cytochrome P450 reductase
DC-PLM	dynamic conformation-aware protein language model
DUF	domain of unknown function
ER	endoplasmic reticulum
FAD	flavin adenine dinucleotide
FiLM	feature-wise linear modulation
FMN	flavin mononucleotide
GH	Glycoside hydrolase
LPMO	lytic polysaccharide monooxygenase
NHMT	N-terminal histidine methyltransferase
POR	NADPH-cytochrome P450 oxidoreductase
PTM	post-translational modification
QM/MM	quantum mechanics/molecular mechanics
SAXS	small-angle X-ray scattering
T5H	tryptamine 5-hydroxylase
TD-DFT	time-dependent density functional theory
TrxA	thioredoxin A
3Di	3D interaction
5-ALA	5-aminolevulinic acid
